# ROS scavenging mitigates TNFα-induced endoplasmic reticulum stress and mitochondrial fragmentation in human airway smooth muscle

**DOI:** 10.1152/function.036.2026

**Published:** 2026-10-01

**Authors:** Debanjali Dasgupta, Sanjana Mahadev Bhat, Gary C. Sieck

**Affiliations:** Department of Physiology and Biomedical Engineering, Mayo Clinic, Rochester, Minnesota, United States

**Keywords:** ER stress, mitochondrial fragmentation, reactive oxygen species, tempol, TNFα

## Abstract

The proinflammatory cytokine tumor necrosis factor alpha (TNFα) mediates airway responses to acute inflammation. Previously, we demonstrated that, in human airway smooth muscle (hASM) cells, TNFα increases reactive oxygen species (ROS) formation. TNFα also selectively activates the inositol-requiring enzyme 1α (pIRE1α^S724^ autophosphorylation) endoplasmic reticulum (ER) stress pathway involving splicing of X-box binding protein 1 (XBP1s) and transcriptionally activates cyclin-dependent kinases 1 and 5 (CDK1 and CDK5), promoting dynamin-related protein 1 (DRP1) phosphorylation at serine 616 (pDRP1^S616^) and mitochondrial fragmentation. In the present study, we hypothesized that in hASM cells, TNFα-induced ROS triggers pIRE1α^S724^/XBP1s ER stress pathway. To test this hypothesis, we examined the impact of the ROS scavenger tempol on TNFα-induced pIRE1α^S724^/XBP1s ER stress pathway and downstream signaling mediating mitochondrial fragmentation. Bronchiolar tissue samples were obtained from six patients with no history of smoking or chronic pulmonary disease. The smooth muscle layer was dissected, and hASM cells were dissociated and randomly assigned to four treatment groups: *1*) vehicle, *2*) vehicle + TNFα (20 ng/mL, 6 h), *3*) tempol (500 μM) only, and *4*) tempol (500 μM) + TNFα (20 ng/mL, 6 h). ROS formation was determined by confocal imaging using MitoSox Red. Mitochondria were labeled with MitoTracker Green and imaged using confocal microscopy. Using Western blot, we demonstrated that tempol reduced cellular ROS formation and mitigated the TNFα-induced increase in pIRE1α^S724^, XBP1s, CDK1/5, pDRP1^S616^ protein levels and reduced mitochondrial fragmentation. These findings support our hypothesis and indicate a role of ROS in mediating TNFα-induced ER stress and mitochondrial fragmentation.

**NEW & NOTEWORTHY** In human airway smooth muscle cells, TNFα induces reactive oxygen species (ROS) generation and promotes endoplasmic reticulum (ER) stress and mitochondrial fragmentation. Importantly, treatment with the ROS scavenger tempol effectively attenuated TNFα-induced oxidative stress, suppressed ER stress and mitochondrial fragmentation, and improved the intrinsic respiratory capacity and functional efficiency of individual mitochondria. These findings identify oxidative stress as a central mediator linking TNFα signaling to ER stress and mitochondrial fragmentation, highlighting tempol as a potential therapeutic strategy.

## Graphical abstract

**Figure:**
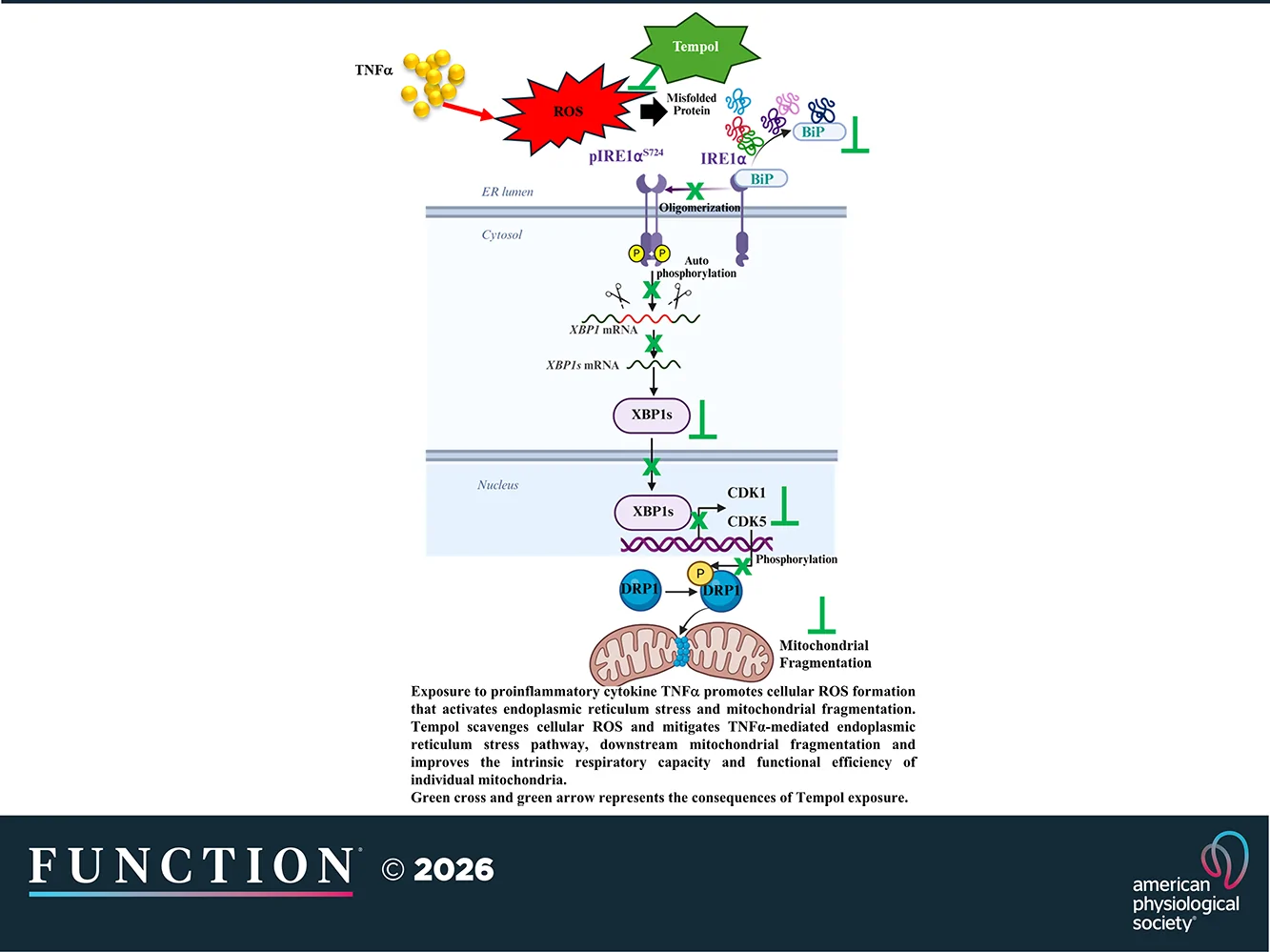

## INTRODUCTION

Acute airway inflammation is a common physiological response to environmental and pathological stimuli, including respiratory infections such as influenza and respiratory syncytial virus (RSV), exposure to allergens, and increasing air pollution (1–5). A key mediator of this inflammatory response is an increase in the proinflammatory cytokine tumor necrosis factor α (TNFα). In airway smooth muscle, TNFα increases force generation, contractile protein expression, and ATP consumption (6–8). The increase in ATP consumption is met by an overall increase in mitochondrial O_2_ consumption rate (OCR) at the expense of increased reactive oxygen species (ROS) formation and oxidative stress (9–11). Although ROS production serves vital physiological functions through redox signaling, excessive or dysregulated ROS production contributes to cellular dysfunction and oxidative stress.

Endoplasmic reticulum (ER) stress occurs when there is an accumulation of unfolded proteins (12–15). Binding immunoglobulin protein (BiP also known as GRP78) is a chaperone protein that binds to unfolded proteins. With an accumulation of unfolded proteins in the ER, BiP dissociates from one or all of three sensor proteins, inositol-requiring enzyme 1 alpha (IRE1α), protein kinase R (PKR)-like endoplasmic reticulum kinase (PERK), and activating transcription factor 6 (ATF6). Activation of each of these sentinel proteins mediates a distinct downstream ER stress signaling cascade (9, 16–18). Previously, we showed that in human airway smooth muscle (hASM), all three ER stress pathways are activated in response to tunicamycin (9, 16–18). However, in hASM, TNFα activates only the IRE1α ER stress pathway by autophosphorylation of IRE1α at serine 724 (pIRE1α^S724^) (9, 16, 17). Downstream, pIRE1α^S724^ promotes splicing of X-box binding protein 1 (XBP1s), which binds to promoter regions of cyclin-dependent kinases 1 and 5 (CDK1 and CDK5), which then mediate phosphorylation of the mitochondrial fission protein dynamin-related protein 1 (DRP1) at serine 616 (pDRP1^S616^) (18). pDRP1^S616^ localizes to mitochondria to mediate mitochondrial fission (18).

**Figure 1.**
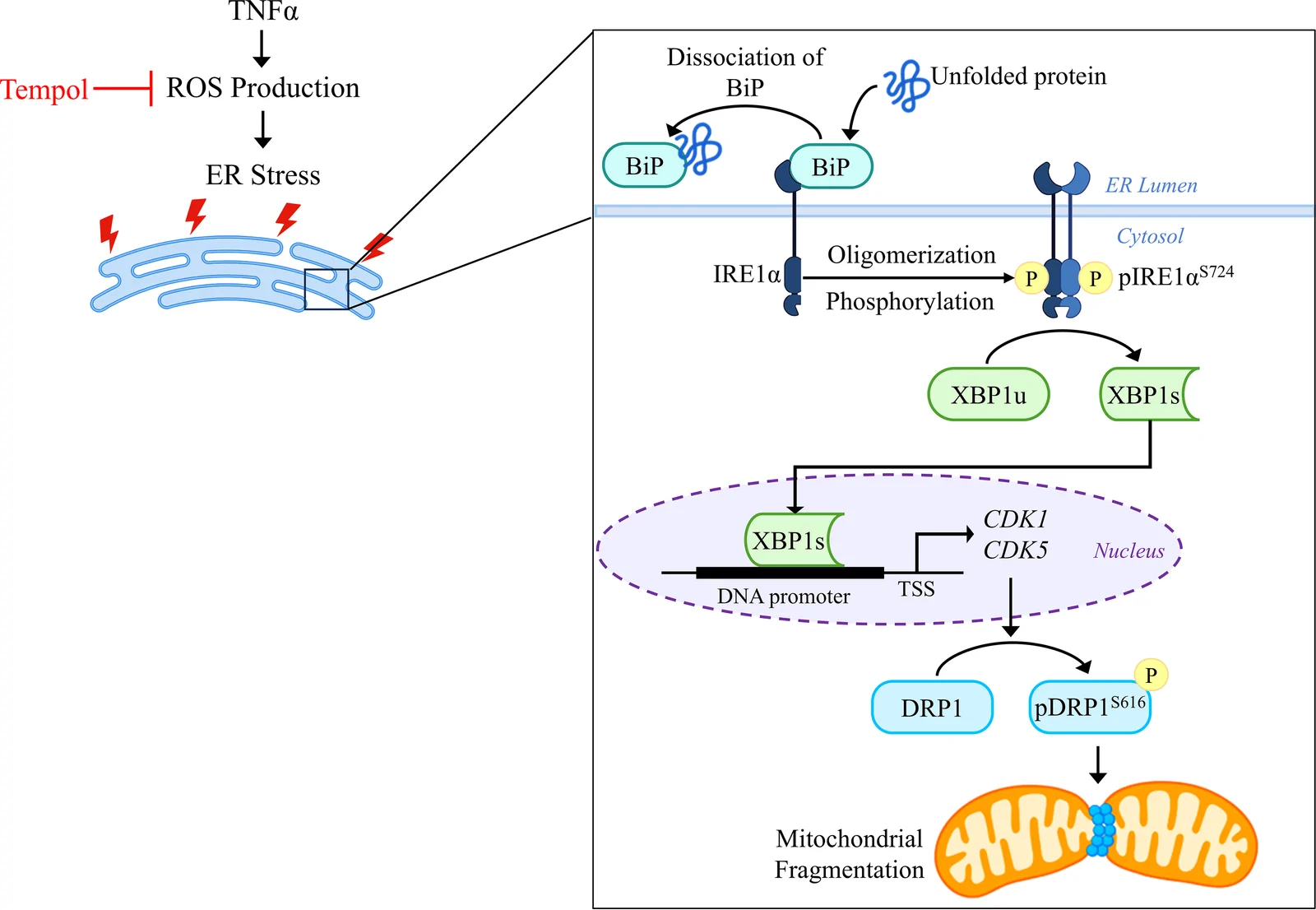
Conceptual framework. In previous studies, we showed that TNFα increases ROS formation and induces endoplasmic reticulum (ER) stress, which activates the unfolded protein response (UPR) through the activation of the UPR sensor protein IRE1α, an ER transmembrane receptor (9). Upon ER stress, IRE1α is activated via oligomerization and autophosphorylation at serine 724 (pIRE1α^S724^) and induces unconventional splicing of XBP1 mRNA by cleaving 26 intronic nucleotides to generate the active transcription factor, spliced XBP1 (XBP1s). XBP1s transcriptionally upregulates the expression of CDK1 and CDK5. CDK1 and CDK5 phosphorylate DRP1 at serine 616 (pDRP1^S616^), which translocates to the mitochondria to promote mitochondrial fragmentation. In the present study, we hypothesize that the ROS scavenger tempol decreases TNFα-induced activation of the pIRE1α^S724^/XBP1s ER stress pathway, thereby reducing transcriptional activation of CDK1 and CDK5, eventually reducing pDRP1^S616^ phosphorylation and mitochondrial fragmentation. Figure created with a licensed version of BioRender.com. ROS, reactive oxygen species; pIRE1α^S724^, autophosphorylation of inositol-requiring enzyme 1α at serine 724; XBP1, X-box binding protein 1.

In a recent study, we showed that by reducing protein unfolding using a chemical chaperone, 4-phenylbutyric acid (4-PBA), TNFα-induced activation of the pIRE1α^S724^/XBP1s ER stress pathway was mitigated in hASM cells (16). In the present study, we hypothesized that TNFα-induced ROS formation is upstream to protein unfolding and thus plays a critical role in the activation of pIRE1α^S724^/XBP1s ER stress pathway and subsequent fragmentation of mitochondria in hASM cells. To test this hypothesis, we used tempol to scavenge TNFα-induced ROS formation in hASM cells (Fig. 1).

## MATERIALS AND METHODS

### Anonymous Patient Selection

The study was reviewed by Mayo Clinic’s Institutional Review Board (IRB No. 16-009655) and considered to be of minimal risk and therefore exempt from further review. All potential patients were consented before surgery in a nonthreatening environment, and a written informed consent was obtained from patients during presurgical evaluation (in the Pulmonary, Oncology, or Thoracic Surgery Clinics). Patient history was provided at the time of tissue acquisition for recording the sex, demographics, smoking status, pulmonary disease status, pulmonary function testing, imaging, comorbidities, and medications. Accordingly, samples were numbered, and none of the patient identifiers were stored or attached to the samples, therefore preventing retrospective patient identification. A total of six (3 females and 3 males; ranging in age from 64 to 72 yr) patients were selected for this study. Patients with chronic lung disease, asthma, any other respiratory diseases, and any recent history of smoking were excluded from the study (Table 1).

**Table 1.** Demographic profile of the patients included in the study

Patient ID	Sex	Age in Years	Current Smoking Status	Respiratory Disease
1	F	64	None	No asthma, COPD, pulmonary fibrosis, or portal hypertension
2	M	64	None	No asthma, COPD, pulmonary fibrosis, or portal hypertension
3	F	64	None	No asthma, COPD, pulmonary fibrosis, or portal hypertension
4	M	72	None	No asthma, COPD, pulmonary fibrosis, or portal hypertension
5	F	70	None	No asthma, COPD, pulmonary fibrosis, or portal hypertension
6	M	69	None	No asthma, COPD, pulmonary fibrosis, or portal hypertension

COPD, chronic obstructive pulmonary disease.

### Dissociation of hASM Cells from Bronchiolar Tissue Samples

**Figure 2.**
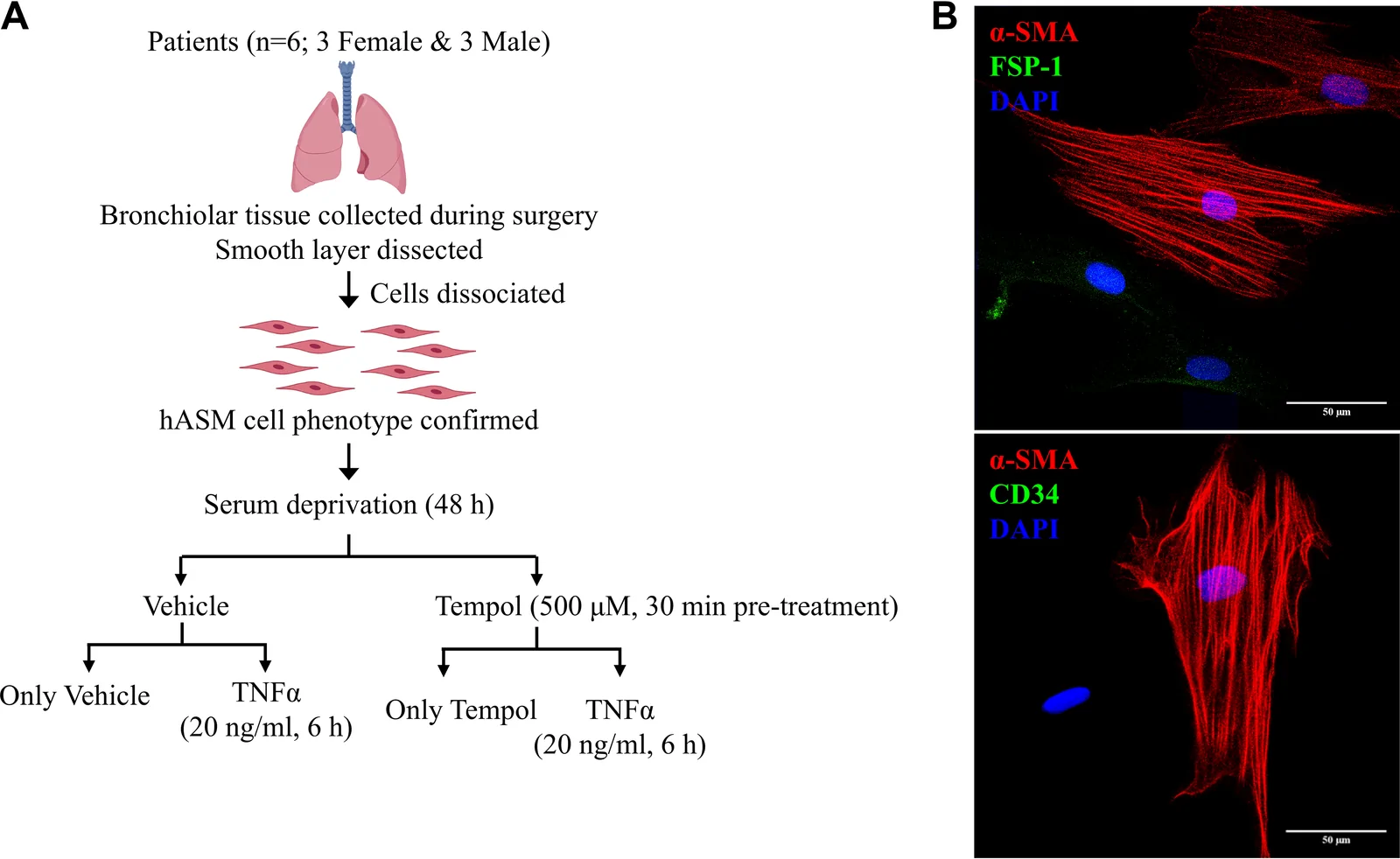
*A*: experimental design. Bronchiolar tissue samples were collected during lung resection surgery from anonymized female and male patients without any history of smoking or respiratory diseases. From each patient, hASM cells were divided into four experimental groups: *1*) vehicle treated, *2*) vehicle + TNFα-treated (20 ng/mL, 6 h), *3*) only tempol (500 μM, 30 min pretreatment) treated, and *4*) tempol (500 μM, 30 min pretreatment) + TNFα (20 ng/mL, 6 h) treated. *B*: phenotyping of dissociated hASM cells. Representative Z-projection image of hASM cells displaying immunoreactivity for α-SMA, FSP-1, and CD34. Dissociated hASM cells also showed morphologically distinct elongated shape, characteristic of hASM cells (scale bar  = 50 μm). Figure created with a licensed version of BioRender.com. α-SMA, α-smooth muscle actin; hASM, human airway smooth muscle.

Bronchiolar tissue samples were obtained from patients undergoing lung resection surgery, evaluated by Clinical Pathology, and determined to be normal. The smooth muscle layer from the third- to sixth-generation bronchi was dissected, and cells were dissociated by enzymatic digestion using papain and collagenase with ovomucoid/albumin separation as per the manufacturer’s instructions (Worthington Biochemical, Lakewood, NJ) and as described previously (19–21). Cells were maintained in phenol red-free DMEM/F-12 medium (Invitrogen, Carlsbad, CA) supplemented with 10% fetal bovine serum (FBS) and 100 U/mL of penicillin/streptomycin, at 37°C (5% CO_2,_ 95% air, pH 7.4). To ensure the use of cells within their optimal phenotypic and metabolic characteristics, cells were only used from 1 to 3 passages. Before treatments, the cells were serum-deprived for 48 h by replacing the growth medium with DMEM/F-12 medium lacking FBS. *Mycoplasma* contamination of cell cultures was monitored by polymerase chain reaction (PCR) on a regular basis. The hASM phenotype of dissociated cells was confirmed by immunoreactivity to α-smooth muscle actin (α-SMA) before experimental use (Fig. 2, *A* and *B*).

### Immunocytochemical Labeling and Phenotyping of Dissociated hASM Cells

Immunocytochemical analysis of α-SMA expression was determined as previously described (9, 22–26). In brief, dissociated cells were plated at a density of ∼10,000 cells/well in a Nunc Lab-Tek II Chamber 8-well multichamber slide (Thermo Fisher Scientific, Rockford, IL), incubated till cell adherence was achieved, and serum-deprived. Cells were fixed with 4% paraformaldehyde in 1X phosphate-buffered saline (PBS) for 10 min at room temperature and washed with 1X PBS. To prevent nonspecific antibody binding, cells were incubated in a blocking buffer (10% normal donkey serum, 0.2% Triton X-100, and 1X PBS) for 1 h at room temperature (RT). Cells were then incubated overnight at 4°C with primary antibodies against the target proteins in a diluent solution (2.5% normal donkey serum, 0.25% sodium azide, 0.2% Triton X-100, 1X PBS). The primary antibodies and the dilutions at which they were used are listed in Table 2. This was followed by incubation for 1 h at RT in a species-specific biotin-conjugated secondary antibody (1:400, diluted in diluent solution; Jackson ImmunoResearch Labs, Cat. No. 711-067-003, RRID: AB_2340595; West Grove, PA), followed by Streptavidin Alexa Fluor 568 (1:200 in PBS; Thermo Fisher Scientific, Cat. No. S-11226, RRID: AB_2315774; Carlsbad, CA) for 30 min at RT. The cells were mounted using Fluoro-Gel II mounting medium containing 4′,6-diamidino-2-phenylindole, dihydrochloride (DAPI; Electron Microscopy Sciences, Hatfield, PA). Based on immunocytochemistry (ICC), the phenotype of dissociated cells was confirmed by the expression of α-SMA (smooth muscle marker). Expression of FSP-1 (fibroblast marker) and CD34 (endothelial cell marker) was also assessed to exclude these cell types. Immunofluorescence and morphometry of dissociated cells were visualized using a Nikon ECLIPSE Ti inverted microscope system (RRID: SCR_021242) with a ×60/1.4 NA oil-immersion objective. The proportion of hASM cells (expressing α-SMA but not FSP-1 or CD34) was determined relative to the total number of dissociated cells (determined from DAPI-labeled nuclei; Fig. 2*B*). Across all patient samples, the fraction of hASM cells solely expressing α-SMA was ∼97% (17, 18, 25–27). These α-SMA-expressing hASM cells were also distinguished by their fusiform shape.

**Table 2.** List of antibodies used in the study

Primary Antibody	Manufacturer	Cat. No.	Application	Dilution
α-SMA	Abcam, Boston, MA	ab5694	ICC	1:500
FSP-1	Abcam, Boston, MA	ab124805	ICC	1:250
CD34	R&D Systems, Pittsburgh, PA	AF4117	ICC	1:500
CDK1	Cell Signaling Technology, Danvers, MA	9116S	Western blot	1:1,000
CDK5	Cell Signaling Technology, Danvers, MA	2506S	Western blot	1:1,000
pDRP1^S616^	Cell Signaling Technology, Danvers, MA	3455S	Western blot	1:500
pIRE1α^S724^	Abcam, Boston, MA	ab124945	Western blot	1:1,000

α-SMA, α-smooth muscle actin; pDRP1^S616^, phosphorylate DRP1 at serine 616; pIRE1α^S724^, autophosphorylation of inositol-requiring enzyme 1α at serine 724.

### Experimental Groups

TNFα (10 µg; Sigma-Aldrich, St. Louis, MO) and tempol (1 g; Santa Cruz Biotechnology, Dallas, TX) were reconstituted in cell culture-grade water to avoid nonspecific effects from the other noninert vehicle. hASM cells isolated from each patient were split into four treatment groups and serum-deprived for 48 h before treatment with *1*) vehicle (water) only, *2*) vehicle (water) + TNFα (20 ng/mL, 6 h), *3*) tempol (500 μM) only, and *4*) tempol (500 μM) + TNFα (20 ng/mL, 6 h) (Fig. 2*A*). The concentration of TNFα and duration of exposure were based on our previous studies, where maximal mitochondrial superoxide production, phosphorylation of pIRE1α^S724^, and splicing of *XBP1* were achieved by 6-h exposure to 20 ng/mL TNFα (9, 16–18).

### Measurement of ROS

hASM cells were plated at a density of ∼15,000 cells/well into 8-well Ibidi microslide plates (Ibidi GmbH, Gewerbehof Gräfelfing, Germany) and incubated to allow cell adherence. Following treatments, hASM cells were loaded with 2.5 μM of MitoSox Red mitochondrial superoxide indicator (Invitrogen, Carlsbad, CA; excitation/emission wavelength: 510/580 nm) using serum-free DMEM/F-12 media and incubated at 37°C for 30 min, followed by extensive washing with Hanks’ balanced salt solution (HBSS) (Thermo Fisher Scientific, Rockford, IL). Subsequently, the same hASM cells were placed in a solution containing 5 μM CM-H_2_DCFDA (chloromethyl-2′,7′-dichlorodihydrofluorescein diacetate, a general oxidative stress indicator; Invitrogen, Carlsbad, CA; excitation/emission wavelength: 495/520 nm) using serum-free DMEM/F-12 media and incubated at 37°C for 10 min, followed by extensive washing with HBSS (Thermo Fisher Scientific, Rockford, IL). The hASM cells were then imaged using a Nikon A1R Confocal Laser Scanning Microscope (RRID: SCR_020317) with a ×60/1.4 NA oil-immersion objective at 12-bit resolution into a 1,024 × 1,024-pixel array. The dynamic range for imaging was set by first scanning unlabeled cells containing no fluorescence signal and then cells treated with the positive control, hydrogen peroxide (H_2_O_2_; 200 μM), which emits the maximum fluorescence signal. A series of 0.5 µm optical slices were acquired for each hASM cell to obtain a Z-stack using the NIS-Elements software (version 5.20.02; RRID: SCR_014329; Nikon Instruments Inc., Melville, NY). Multiple hASM cells were visualized within a single microscopic field. The three-dimensional (3-D) images obtained were deconvolved using the automatic deconvolution algorithm on NIS-Elements software (Modified Richardson-Lucy method; Point Scan Confocal modality; Nikon Instruments Inc.; version 5.20.02; RRID: SCR_014329) (24, 28–30). Deconvolution enhances the signal-to-noise ratio in the images, thereby improving contrast and edge detection. The voxel dimensions of each deconvolved optical slice were 0.207 × 0.207 × 0.5 µm. After deconvolution, the boundaries of each hASM cell were delineated, a region of interest (ROI) was delineated, and the mean fluorescence intensity was measured using the NIS-Elements software (version 5.20.02; RRID: SCR_014329; Nikon Instruments Inc., Melville, NY).

### Measurement of OCR

hASM cells were plated on Seahorse XFe24 cell culture microplates (Agilent Technologies, Santa Clara, CA) as recommended by the manufacturer for optimal adherence and growth of mammalian cells. The hASM cells were then incubated to allow for cell adherence and formation of a confluent monolayer and then serum-deprived for 48 h. Following treatment, OCR was measured using the mitochondrial stress test assay performed using the Agilent Seahorse XFe24 Cell Analyzer (RRID: SCR_019539; Agilent Technologies, Santa Clara, CA) as per the manufacturer’s instructions and as described previously (25, 27, 31). On the day of the assay, the serum-free media was replaced with the recommended Seahorse XF DMEM base medium (Agilent, Santa Clara, CA), supplemented with 10 mM glucose, 1 mM sodium pyruvate, and 2 mM glutamine at pH 7.4 and maintained in a 37°C CO_2_-free incubator for 1 h. Before the assay, the Seahorse XFe24 FluxPak sensor cartridge was hydrated using Seahorse XF Calibrant Solution for 12–24 h. The electron transport chain (ETC) complex inhibitors and FCCP were loaded into the injection ports on the sensor cartridge at optimal concentrations determined for hASM cells, which were 1 µM oligomycin (ATP synthase inhibitor), 1.25 µM FCCP (mitochondrial uncoupler), and 1 µM antimycin A (complex III inhibitor) with 1 µM rotenone (complex I inhibitor) (32, 33). This allowed for the determination of ATP-linked respiration and maximum respiration. All OCR measurements were normalized to total adherent cell count obtained at the beginning of the assay (24, 34). The total number of cells within each well was obtained by in situ cell counting before every mitochondrial stress test using 1 µg/mL Hoechst 33342 Solution (Invitrogen, Carlsbad, CA; excitation/emission wavelength: 361/486 nm) and Cytation 5 Cell Imaging Multi-Mode Reader (RRID: SCR_019732; BioTek, Winooski, VT). All data were analyzed using Seahorse Wave Software (version 2.6.3; RRID: SCR_014526; Agilent Technologies, Santa Clara, CA). In separate experiments, mitochondrial volume density was quantified as described in *Analysis of Mitochondrial Volume Density*, and all OCR measurements were also normalized to obtain mitochondrial-specific measurements. hASM cells from the same six patients were used for OCR measurement (4 wells/patient).

### Labeling and Confocal Imaging of Mitochondria

hASM cells were plated at a density of ∼15,000 cells/well into 8-well Ibidi microslide plates (Ibidi GmbH, Gewerbehof Gräfelfing, Germany) and incubated to allow cell adherence. hASM cells in each treatment group were loaded with 200 nM MitoTracker Green FM (Invitrogen, Carlsbad, CA; excitation/emission wavelength: 490/516 nm), in serum-free DMEM/F-12 media, prewarmed, at 37°C for 15 min, followed by extensive washing with HBSS (Thermo Fisher Scientific, Rockford, IL). Mitochondria in hASM cells were imaged using the same confocal microscope system used for superoxide measurements as described earlier (24, 29, 30, 35). The dynamic range for imaging was set by first scanning a region containing no fluorescence signal and then a second region containing maximum fluorescence signal. A series of 0.5 µm optical slices were acquired for each hASM cell to obtain a Z-stack using the NIS-Elements software (version 5.20.02; RRID: SCR_014329; Nikon Instruments Inc., Melville, NY).

### Analysis of Mitochondrial Volume Density

The 3-D images obtained were deconvolved and processed as described earlier for mitochondrial superoxide measurement. After deconvolution, the boundaries of each hASM cell were delineated, and ROIs were defined using ImageJ-Fiji software (https://imagej.nih.gov/ij/; version 1.53t, RRID: SCR_002285; NIH, Public Domain). Using the mitochondrial analyzer plugin (https://github.com/AhsenChaudhry/Mitochondria-Analyzer) available on ImageJ, the Z-stacks were then processed for background correction and ridge filter detection (35–38). The mitochondria within hASM cells were identified by thresholding to create a binary image and then skeletonized for morphometric analysis. Using the thresholded binary image, total mitochondrial volume was measured by the mitochondrial analyzer plugin, where the number of voxels containing fluorescently labeled mitochondria within an ROI was determined (31, 35, 39). Mitochondrial volume density was calculated as the ratio of mitochondrial volume within an ROI to the total volume of the defined ROI (mean cross-sectional area of the ROI from all optical slices × total number of slices × 0.5 µm) and represented as a percentage of the volume of the ROI. Based on an a priori power analysis of variance in mitochondrial volume density measurements in untreated hASM cells, *n* = 10 individual hASM cells per bronchiolar sample (from 6 patients) were included in the analysis.

### Analysis of Mitochondrial Complexity Index

From the 3-D images of MitoTracker-labeled hASM cells, mitochondrial morphological parameters, including mitochondrial volume and mitochondrial surface area, were measured within the ROI. The extent of mitochondrial fragmentation was assessed by calculating the mitochondrial complexity index (MCI) using the following equation (18, 27, 31, 40):

$$MCI=\frac{SA^{3}}{16\pi^{2}V^{2}},$$

where SA is the total mitochondrial surface area measured within the ROI, and V is the total volume occupied by the mitochondria within the ROI. More fragmented mitochondria exhibit lower surface area, higher volume, and thus lower MCI.

### Protein Extraction and Western Blot for Target Protein Expression

Protein samples were extracted from hASM cells in each treatment group using 1X cell lysis buffer (Cell Signaling Technology, Danvers, MA) supplemented with protease (cOmplete, protease inhibitor cocktail; Roche, Basel, Switzerland) and phosphatase inhibitors (PhosSTOP; Roche, Basel, Switzerland). The concentration of the extracted protein was measured by detergent compatible (DC) protein assay (Bio-Rad, Hercules, CA), following the manufacturer’s instructions. For each Western blot, 40 μg of total protein from each sample was denatured at 95°C for 5 min in 1X Laemmli sample buffer with 5% β-mercaptoethanol and loaded into 4%–20% Criterion TGX Stain-Free protein gel (Bio-Rad, Hercules, CA). Once separated, the total protein loaded into each lane was visualized using the Bio-Rad ChemiDoc MP imaging system (RRID:SCR_019037; Hercules, CA). The amount of total protein loaded in each lane was measured from the stain-free gel by quantifying the cumulative intensities of all individual protein bands in the lane using the Image Lab software (version 6.0.1; RRID:SCR_014210; Bio-Rad, Hercules, CA). The separated protein bands were then transferred to a polyvinylidene difluoride (PVDF) membrane (0.2 μm; Bio-Rad, Hercules, CA) using the Bio-Rad Trans Blot Turbo system (RRID:SCR_023156; Hercules, CA). The Western blot membrane from the same experiment where samples, conditions, and time points are the same, was divided horizontally into sections to probe for multiple proteins of different molecular weights simultaneously using distinct antibodies, to avoid gel to gel variation, any batch effect, and the problem of restripping and reprobing cycles. Following transfer, the membranes were blocked using 5% nonfat dry milk for 1 h at room temperature, followed by overnight incubation at 4°C in primary antibody to recognize and bind to the protein of interest (Table 2). The membranes were incubated with species-specific horseradish peroxidase (HRP)-conjugated secondary antibody (1:7,500; Jackson ImmunoResearch Labs, Cat. No. 115-035-003, RRID: AB_10015289) for 1 h at room temperature to detect the primary antibody and amplify the signal for detection. Bands were developed by incubating the membranes in chemiluminescent SuperSignal West Dura Extended Duration Substrate (Thermo Fisher Scientific, Rockford, IL) for 3 min and visualized using the ChemiDoc MP imaging system. The band intensity of the targeted protein band was then analyzed using the Image Lab software. The band intensity of the protein of interest was normalized to the total protein loaded in their respective lanes. In a previous study, quantification of total protein per band was verified by systematic dilution of the lysate, thereby changing the amount of protein loaded, then probing the blot with RPS16 (18). We confirmed that levels of RPS16 corresponded to the total protein loaded in the lane. Importantly, normalization to total protein per lane makes no assumptions regarding the lack of an effect on housekeeping proteins. All antibodies were validated before use, as described in previous studies (9, 18, 22). All gel and full blot images are provided in the Supplemental Figs. S1–S3.

### RNA Extraction, cDNA Preparation, qPCR for Target Gene Expression

Total RNA was extracted from treated and untreated hASM cells using the RNeasy extraction kit (Qiagen, Hilden, Germany) as per the manufacturer’s instructions. In brief, hASM cells were lysed and subjected to ethanol-mediated extraction of RNA. Genomic DNA was removed by an on-column DNase treatment. Extracted RNA samples were quantified using a Thermo Scientific NanoDrop One/OneC Microvolume UV–Vis Spectrophotometer (RRID:SCR_023005; Thermo Fisher Scientific, Rockford, IL). A total of 500 ng of RNA was used for complementary DNA (cDNA) synthesis using the high-capacity cDNA reverse transcription kit (Invitrogen, Carlsbad, CA) following the manufacturer’s instructions. qPCR was performed using 500 ng of cDNA for *XBP1s* using SYBR Green (Cat. No. 04707516001, Roche, Basel, Switzerland) and Bio-Rad T100 Thermal Cycler (RRID:SCR_021921; Hercules, CA). RPS16 was used as the housekeeping gene. No-template negative controls and melting dissociation curves were run for all reactions to exclude cross contamination. The primer sequences used for *XBP1s* and *RPS16* are listed in Table 3.

**Table 3.** List of primers used in the study

Gene Name	Primer Name	Primer Sequence
*RPS16*	RPS16-F	5′- GTCTGTGCAGGTCTTCGGACGC-3′
	RPS16-R	5′- GACCATTGCCGCGTTTGCAGTG-3′
*XBP1s*	XBP1s-F	5′- TCTGCTGAGTCCGCAGCAGG-3′
	XBP1s-R	5′- CTCTAAGACTAGAGGCTTGG-3′

XBP1, X-box binding protein 1.

### Validation of the Antibodies and Primers

All primers and antibodies were validated before use for PCR and Western blot, respectively. Primers were validated using a no-template (no DNA) negative control and melting curve analysis. Antibodies were validated either in our previous studies (17, 18, 26) or by the manufacturers as mentioned in their datasheet.

### Statistical Analysis

Sex is an important biological variable; therefore, hASM cells for all experiments were dissociated from bronchial tissue samples obtained from three female and three male patients (distinguished by symbol shape). In previous studies, we found no evidence for sex differences in TNFα-induced pIRE1α^S724^ and *XBP1s* or downstream signaling (18). Accordingly, this study was not powered to detect sex differences as a major outcome variable; however, the study was balanced for sex as a random variable in the statistical analysis. All experiments were conducted using the same six patients to maintain consistency and reduce variability. Importantly, hASM cells from the same patient and passage (1–3 passages) were randomly assigned to each treatment group, and all groups were processed at the same time and under the same experimental conditions to exclude any batch effects. Therefore, hASM cells from each patient (designated by color) served as their own control. The baseline outcome measures varied across patient samples; however, patient-to-patient comparisons were beyond the scope of the present study. Sample size (*n*) represents the number of bronchial samples (patients), and the *n* used for each experiment is provided in the figure legends. The required minimum number of patients was determined by a power analysis of primary outcome measures. The expected effect size was calculated with an a priori physiologically relevant difference of 20% and equal variance, with sample size estimated using *d* = 1.4, α = 0.05, and β = 0.8 (10 cells/patient; 6 patients). The experiments were designed such that the comparisons were made within a patient. Statistical analysis was performed using GraphPad Prism 10 (version: 10.4.2, RRID: SCR_002798; San Diego, CA). Normal distribution of the data was confirmed using the Shapiro–Wilk test. For all experiments, statistical analyses were performed using mixed-effects analysis, considering “patient” as a random effect and “treatment” as a fixed effect. Statistical significance was concluded if *P* < 0.05, indicated by * in the figures.

## RESULTS

### Phenotype of Dissociated hASM Cells

Consistent with our previous studies (16, 18, 25–27), almost all cells dissociated from the smooth muscle layer dissected from third- to sixth-generation bronchi were solely immunoreactive to α-SMA (∼97%), indicating that they were hASM cells with the remainder being fibroblasts (Fig. 2*B*). These hASM cells were also larger and displayed a characteristic elongated shape that distinguished them from other cells. In all experiments involving confocal imaging, the distinct morphological features of the cells were used to identify hASM cells.

### Tempol Decreases TNFα-Induced Superoxide Production

**Figure 3.**
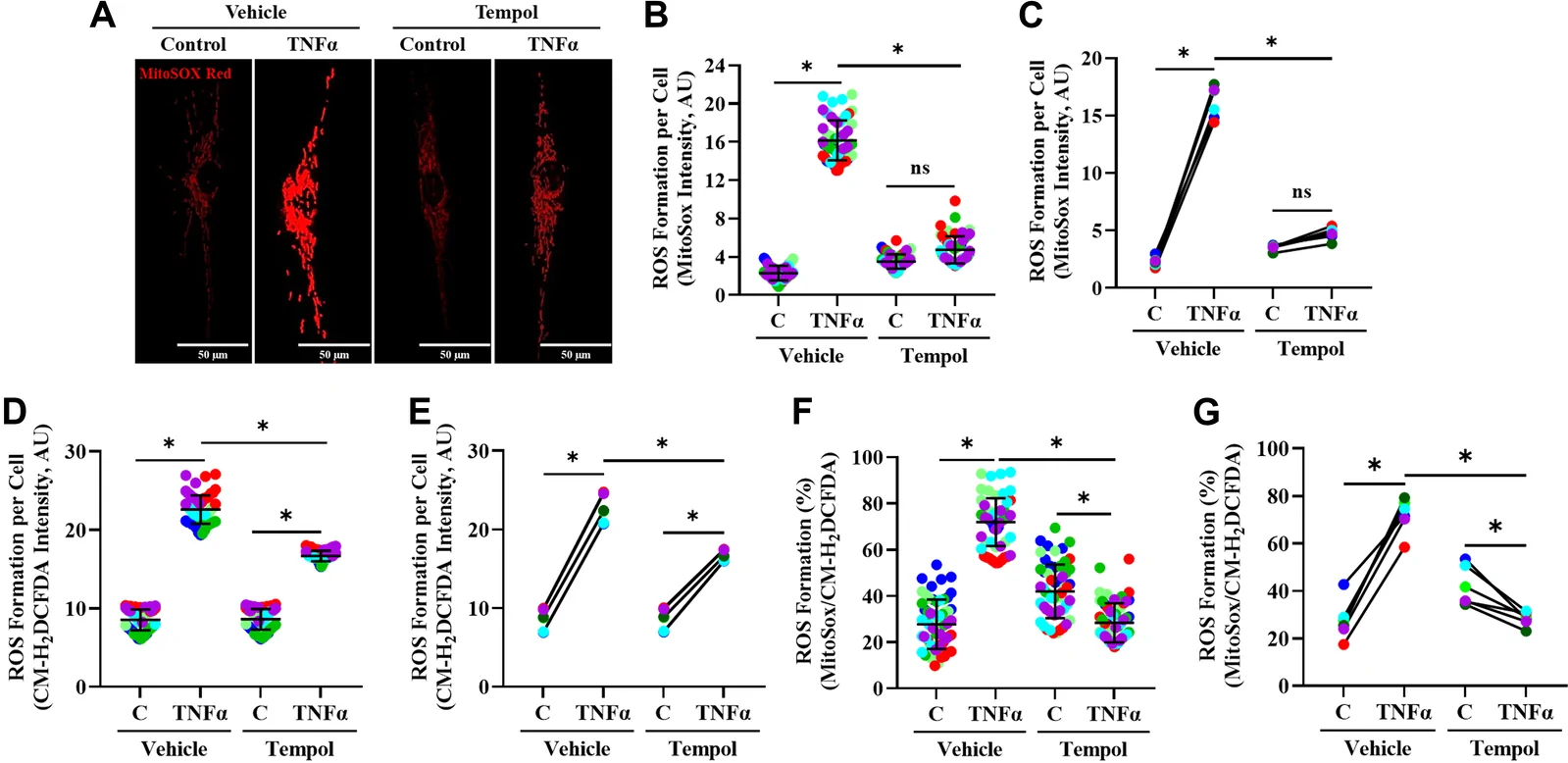
Tempol mitigates TNFα-induced superoxide production. *A*: representative maximum intensity Z-projection image shows hASM cell loaded with MitoSox Red to visualize the production of mitochondrial superoxide (scale bar = 50 μm). Mitochondrial superoxide production was measured as change in fluorescent intensity using three-dimensional (3-D) reconstructed Z-stack images of fluorescently labeled mitochondria using ImageJ in hASM cells treated with vehicle, TNFα (20 ng/mL, 6 h), only tempol (500 μM, 30 min pretreatment), and TNFα (20 ng/mL, 6 h) with tempol (500 μM, 30 min pretreatment). *B* and* C*: TNFα increased mitochondrial superoxide when compared with controls (*P < 0.05). Pretreatment with tempol decreased TNFα-mediated superoxide production significantly (*P < 0.05). Scatter plot (*B*) and symbols-and-lines plot (*C*) shows mean fluorescent intensity of MitoSox per cell from 6 patients (3 females and 3 males; 10 cells/patient) per treatment group. Statistical significance was determined based on a mixed-effects analysis. *D* and* E*: TNFα increased total cellular superoxide when compared with controls (*P < 0.05). Pretreatment with tempol significantly decreased TNFα-mediated superoxide production (*P < 0.05). Scatter plot (*D*) and symbols-and-lines plot (*E*) shows mean fluorescent intensity of CM-H_2_DCFDA per cell from 6 patients (3 females and 3 males; 10 cells/patient) per treatment group. *F* and* G*: TNFα increased MitoSox to CM-H_2_DCFDA ratio, when compared with controls (*P < 0.05). Pretreatment with tempol significantly decreased TNFα-mediated MitoSox to CM-H_2_DCFDA ratio (*P < 0.05). Scatter plot (*F*) and symbols-and-lines plot (*G*) shows MitoSox to CM-H_2_DCFDA ratio from 6 patients (3 females and 3 males; 10 cells/patient) per treatment group. Each color represents results from one bronchial sample. Data are presented as means ± SD in scatter plot. Statistical analyses were based on measurements from *n* = 6 (3 females and 3 males) patients using mixed-effects analysis. hASM, human airway smooth muscle.

Superoxide levels were measured in hASM cells in all treatment groups using MitoSox and DCFDA (Fig. 3). Compared with vehicle-treated hASM cells, TNFα significantly increased ROS generation in hASM cells from all six patients (Fig. 3; *P* < 0.05). Mitochondrial ROS levels were measured in hASM cells in all treatment groups using MitoSox, and TNFα significantly increased cellular ROS generation by approximately fivefold compared with vehicle-treated hASM cells (Fig. 3, *B* and *C*). The TNFα-induced increase in mitochondrial ROS was significantly mitigated by treatment with tempol (Fig. 3, *A*–*C*; *P* < 0.05). Total cellular ROS levels were measured in hASM cells in all treatment groups using CM-H_2_DCFDA (Fig. 3, *D* and *E*). Compared with vehicle-treated hASM cells, TNFα significantly increased cellular ROS generation by ∼2.5-fold (Fig. 3, *D* and *E*; *P* < 0.05). The TNFα-induced increase in cellular ROS was significantly mitigated by treatment with tempol (Fig. 3, *D* and *E*; *P* < 0.05). As demonstrated by the ratio of MitoSox versus DCFDA, TNFα significantly increased the mitochondrial ROS compared to cellular ROS, and tempol significantly mitigated that increase (Fig. 3, *F* and *G*; *P* < 0.05). No significant difference was observed between vehicle-treated hASM cells and cells treated only with tempol.

### Tempol Mitigates TNFα-Induced Phosphorylation of pIRE1α^S724^

**Figure 4.**
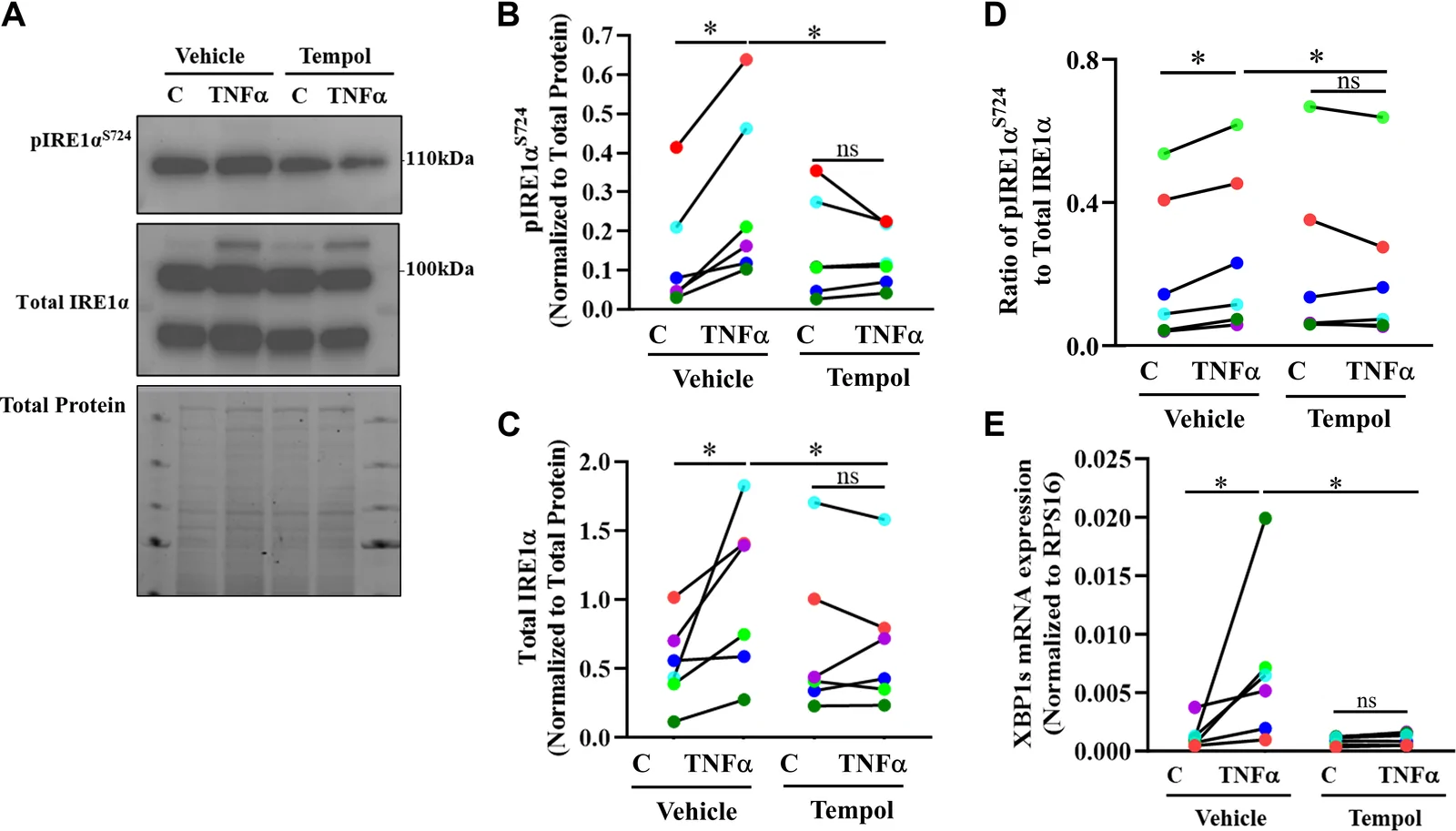
Tempol mitigates TNFα-induced pIRE1α^S724^/XBP1s endoplasmic reticulum (ER) stress. *A*: representative Western blot shows the expression of pIRE1α^S724^ and total IRE1α in hASM cells treated with vehicle, TNFα (20 ng/mL, 6 h), only tempol (500 μM, 30 min pretreatment), and TNFα (20 ng/mL, 6 h) with tempol (500 μM, 30 min pretreatment). *B*: TNFα increased pIRE1α^S724^ phosphorylation, *C*: total IRE1α expression and *D*: ratio of pIRE1α^S724^ to total IRE1α in hASM cells compared with control (*P < 0.05). Pretreatment with tempol reduces the TNFα-induced pIRE1α^S724^ phosphorylation (*B*), total IRE1α expression (*C*) and ratio of pIRE1α^S724^ to total IRE1α (*D*) (*P < 0.05). Symbols-and-lines plot shows data from 6 patients (3 females and 3 males) per treatment group. *E*: TNFα increased spliced *XBP1* (*XBP1s*) mRNA expression levels in hASM cells compared with control (*P < 0.05), pretreatment with tempol reduced the TNFα-induced increase in *XBP1s* (*P < 0.05). Symbols-and-lines plot shows *XBP1s* from 6 patients (3 females and 3 males) per treatment group. Each color represents results from one bronchial sample (patient). Data are presented as symbols-and-lines plot. Statistical analyses were based on measurements from *n* = 6 (3 females and 3 males) patients using mixed-effects analysis. hASM, human airway smooth muscle; pIRE1α^S724^, autophosphorylation of inositol-requiring enzyme 1α at serine 724; XBP1, X-box binding protein 1.

Consistent with previous studies (9, 16, 18), 6 h treatment with TNFα increased the autophosphorylation of pIRE1α^S724^ by ∼1.7-fold compared to vehicle-treated hASM cells (Fig. 4, *A* and *B*, Supplemental Fig. S1; *P* < 0.05). Treatment with tempol significantly reduced TNFα-induced autophosphorylation of pIRE1α^S724^ compared to cells treated only with TNFα (Fig. 4, *A* and *B*, Supplemental Fig. S1; *P* < 0.05). No significant difference in pIRE1α^S724^ was observed between vehicle-treated hASM cells and cells treated only with tempol. As a result of ER stress-mediated transcriptional regulation, total IRE1α expression was also increased after 6 h treatment with TNFα by 1.9-fold compared to vehicle-treated hASM cells, which is also consistent with our previously published data (Fig. 4, *A* and *C*, Supplemental Fig. S1; *P* < 0.05) (18). Treatment with tempol significantly reduced TNFα-induced total IRE1α expression. No significant difference in total IRE1α was observed between vehicle-treated hASM cells and cells treated only with tempol. Along with the abundance of IRE1α, 6 h treatment with TNFα increased the ratio of pIRE1α^S724^ to total IRE1α by 1.3-fold, indicating robust activation of the IRE1α-mediated ER stress pathway (Fig. 4, *A* and *D*, Supplemental Fig. S1; *P* < 0.05). Tempol reduced the ratio of pIRE1α^S724^ to total IRE1α compared to TNFα (Fig. 4, *A* and *D*, Supplemental Fig. S1; *P* < 0.05).

### Tempol Decreases TNFα-Mediated XBP1 mRNA Splicing

To determine the extent of *XBP1* mRNA splicing, relative (normalized to RPS16) *XBP1s* mRNA expression was measured using RT-qPCR (Fig. 4*E*). Across all patients, *XBP1s* mRNA significantly increased by ∼2.2-fold after 6 h treatment with TNFα compared to vehicle-treated hASM cells (Fig. 4*E*; *P* < 0.05). Treatment with tempol significantly reduced TNFα-induced *XBP1s* mRNA expression compared to the hASM cells treated with TNFα only (Fig. 4*E*; *P* < 0.05). No significant difference in *XBP1s* mRNA expression was observed between vehicle-treated hASM cells and cells treated only with tempol.

### Tempol Decreases Expression of CDK1 and CDK5 in hASM Cells

**Figure 5.**
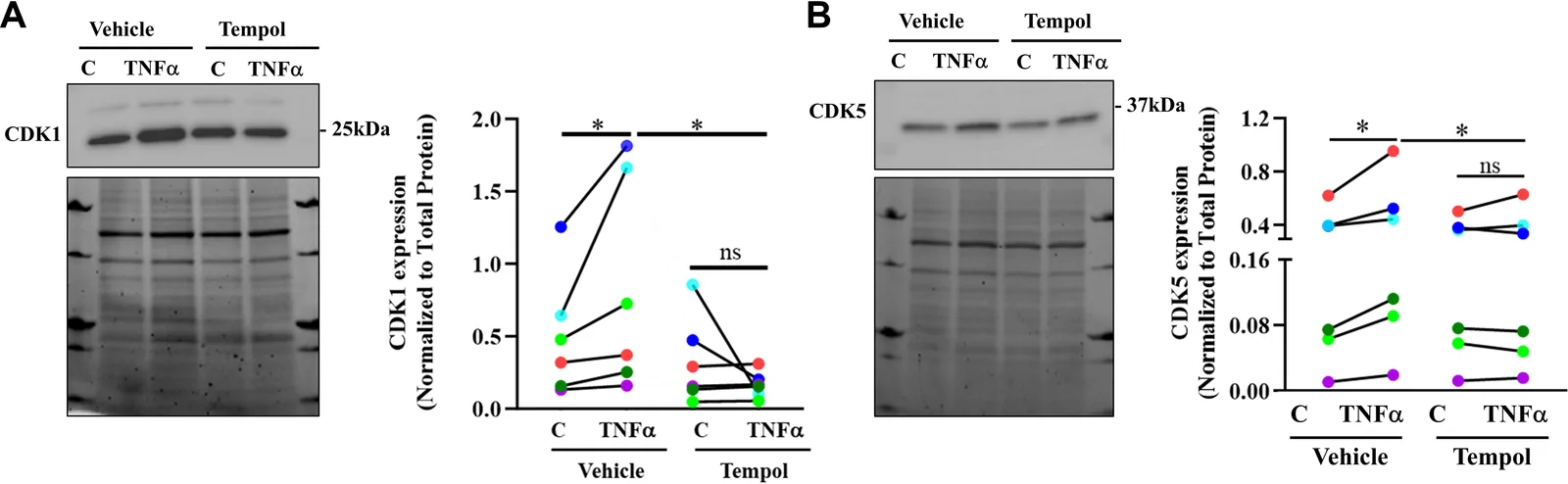
Tempol mitigates TNFα-induced expression of CDK1 and CDK5 in hASM cells. *A*: representative Western blot shows the expression of CDK1 in hASM cells treated with vehicle, TNFα (20 ng/mL, 6 h), only tempol (500 μM, 30 min pretreatment), and TNFα (20 ng/mL, 6 h) with tempol (500 μM, 30 min pretreatment). TNFα treatment increased CDK1 expression (*P < 0.05), whereas pretreatment with tempol decreased TNFα-induced CDK1 expression (*P < 0.05). Symbols-and-lines plot shows CDK1 expression from 6 patients (3 females and 3 males) per treatment group. *B*: representative Western blot shows the expression of CDK5 in hASM cells treated with vehicle, TNFα (20 ng/mL, 6 h), only tempol (500 μM, 30 min pretreatment), and TNFα (20 ng/mL, 6 h) with tempol (500 μM, 30 min pretreatment). Similar to CDK1, TNFα treatment increased CDK5 expression (*P < 0.05), whereas pretreatment with tempol decreased TNFα-induced CDK5 expression (*P < 0.05). Symbols-and-lines plot shows CDK5 expression from 6 patients (3 females and 3 males) per treatment group. Each color represents results from one bronchial sample (patient). Data are presented as symbols-and-lines plot. Statistical analyses were based on measurements from *n* = 6 (3 females and 3 males) patients using mixed-effects analysis. hASM, human airway smooth muscle.

In a previous study, we demonstrated that XBP1s binds to the promoters of *CDK1* and *CDK5* and transcriptionally targets *CDK1* and *CDK5* gene expression in hASM cells (18). Consistent with our previous finding, exposure to TNFα for 6 h increased CDK1 and CDK5 expression in hASM cells from each patient sample, with an overall 1.7-fold increase in CDK1 and 1.4-fold increase in CDK5 (Fig. 5, *A* and *B*, Supplemental Fig. S2, *A*–*D*; *P* < 0.05). Treatment with tempol significantly reduced the TNFα-induced increase in CDK1 expression by ∼4.2-fold compared to cells treated with TNFα only (Fig. 5*A*, Supplemental Fig. S2, *A* and *B*; *P* < 0.05). Similarly, tempol treatment reduced the TNFα-induced increase in CDK5 expression by ∼1.3-fold (Fig. 5*B*, Supplemental Fig. S2, *C* and *D*; *P* < 0.05). No significant difference was observed in CDK1 and CDK5 between vehicle-treated hASM cells and cells treated only with tempol.

### Tempol Decreases Phosphorylation of pDRP1^S616^

**Figure 6.**
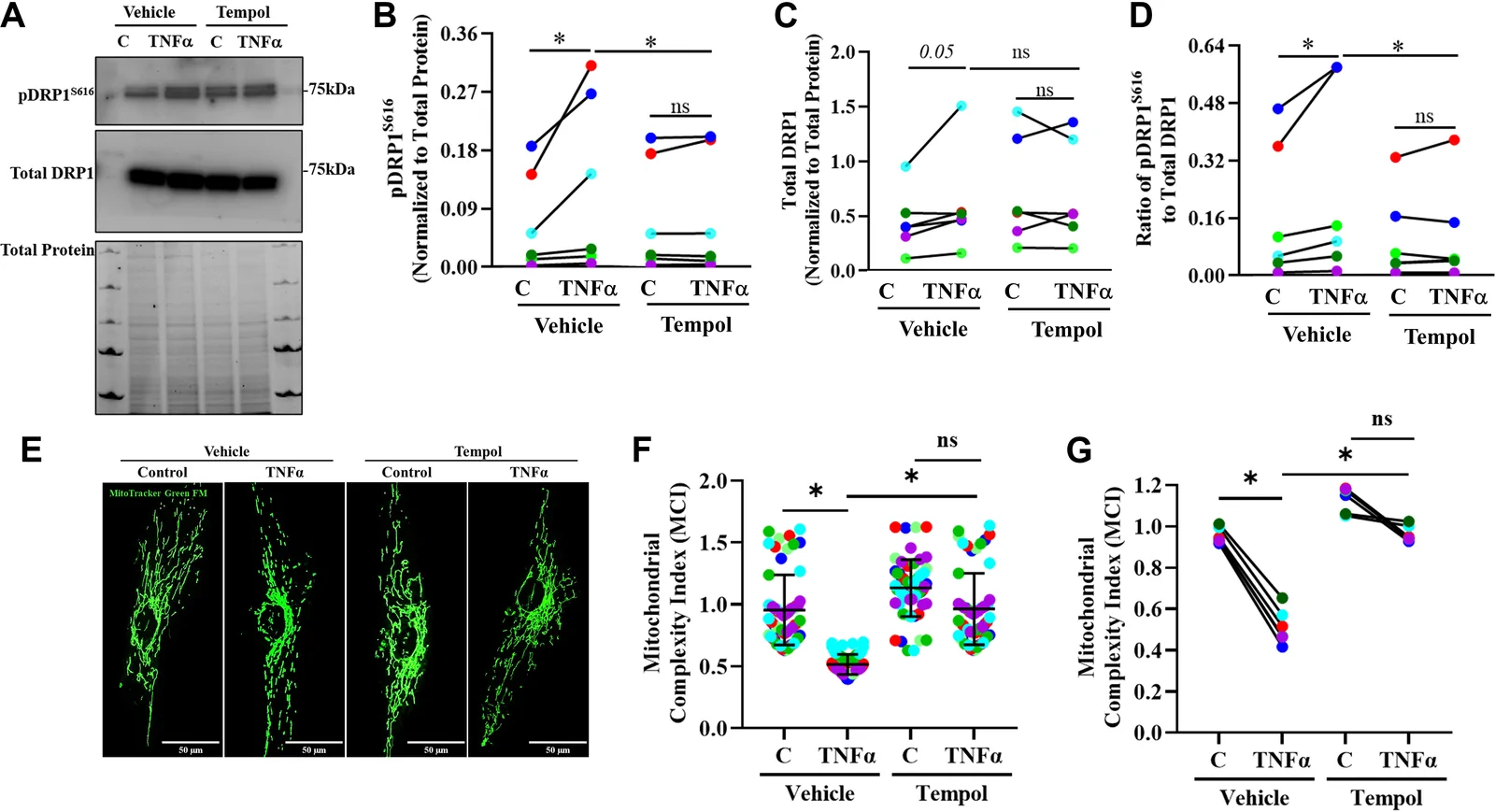
Tempol reduces mitochondrial fragmentation in hASM cells. *A*: representative Western blot shows the expression of pDRP1^S616^ and total DRP1 in hASM cells treated with vehicle, TNFα (20 ng/mL, 6 h), only tempol (500 μM, 30 min pretreatment), and TNFα (20 ng/mL, 6 h) with tempol (500 μM, 30 min pretreatment). TNFα treatment increased (*B*) pDRP1^S616^ expression (*P < 0.05), whereas pretreatment with tempol decreased TNFα-induced pDRP1^S616^ expression (*P < 0.05). Furthermore, tempol-mediated decrease in pDRP1^S616^ was comparable to control (*P < 0.05). Symbols-and-lines plot shows pDRP1^S616^ expression from 6 patients (3 females and 3 males) per treatment group. *C*: total DRP1 expression was increased, although not significantly, after TNFα treatment and was unchanged after Tempol treatment. *D*: pDRP1^S616^ to total DRP1 ratio was increased after TNFα treatment, whereas pretreatment with tempol decreased TNFα-induced pDRP1^S616^ to total DRP1 ratio (*P < 0.05). Furthermore, tempol-mediated decrease in pDRP1^S616^ was comparable to control (*P < 0.05). Symbols-and-lines plot shows the data from 6 patients (3 females and 3 males) per treatment group. *E*: representative maximum intensity Z-projection image shows hASM cell loaded with MitoTracker Green FM to visualize mitochondria (scale bar = 50 μm). Mitochondrial complexity index (MCI) was measured using three-dimensional (3-D) reconstructed Z-stack images of fluorescently labeled mitochondria using ImageJ in hASM cells treated with TNFα (20 ng/mL, 6 h), TNFα (20 ng/mL, 6 h) with tempol (500 μM, 30 min pretreatment), and untreated (control). *F* and* G*: MCI was decreased in TNFα-treated cells when compared with controls (*P < 0.0001). Pretreatment with tempol increases MCI (*P < 0.05), indicative of decreased mitochondrial fission. Scatter plot (*F*) and symbols-and-lines plot (*G*) show MCI from 6 patients (3 females and 3 males; 10 cells/patient) per treatment group. Each color represents results from one bronchial sample (patient). Data are presented as means ± SD in scatter plot. Statistical analyses were based on measurements from *n* = 6 (3 females and 3 males) patients using mixed-effects analysis. hASM, human airway smooth muscle.

Consistent with previous studies (18, 30), we observed an ∼1.9-fold increase in phosphorylation of pDRP1^S616^ after 6 h TNFα treatment compared to vehicle-treated hASM cells (Fig. 6, *A* and *B*, Supplemental Fig. S3; *P* < 0.05). Treating hASM cells with tempol reduced the TNFα-induced increase in pDRP1^S616^ phosphorylation compared to hASM cells treated only with TNFα (Fig. 6*A*, Supplemental Fig. S3; *P* < 0.05). No significant difference in pDRP1^S616^ phosphorylation was observed between vehicle-treated hASM cells and cells treated only with tempol. Consistent with our previous finding (18), total DRP1 expression was also increased after 6 h treatment with TNFα by 1.3-fold compared to vehicle-treated hASM cells (Fig. 6, *A* and *C*, Supplemental Fig. S3; *P* = 0.05) (18). Along with the abundance of DRP1, 6 h treatment with TNFα increased the ratio of pDRP1^S616^ to total DRP1 by 1.4-fold (Fig. 6, *A* and *D*, Supplemental Fig. S3; *P* < 0.05). Treatment with tempol had no effect on TNFα-induced total DRP1 expression; however, tempol significantly reduced the ratio of pDRP1^S616^ to total DRP1 by ∼2.1-fold (Fig. 6, *A* and *D*, Supplemental Fig. S3; *P* = 0.05). This finding signifies that TNFα-mediated DRP1 expression is mediated by other TNFα-responsive transcriptional regulators not related to pIRE1α^S724^/XBP1s ER stress pathway.

### Tempol Decreases TNFα-Induced Mitochondrial Fragmentation

An increase in pDRP1^S616^ is associated with mitochondrial fragmentation as assessed in hASM cells using confocal imaging and calculation of MCI. Consistent with previous studies (18, 30, 31), MCI was significantly decreased (mitochondria more fragmented) by ∼2-fold in hASM cells treated with TNFα for 6 h compared to vehicle-treated control hASM cells (Fig. 6, *E* and *F*; *P* < 0.0001). Treatment with tempol significantly reduced the TNFα-induced decrease in MCI (less mitochondrial fragmentation) compared to cells treated only with TNFα (Fig. 6, *E* and *F*; *P* < 0.0001). No significant difference in MCI was observed between vehicle-treated hASM cells and cells treated only with tempol.

### Tempol Decreases TNFα-Induced Increase in Mitochondrial OCR

**Figure 7.**
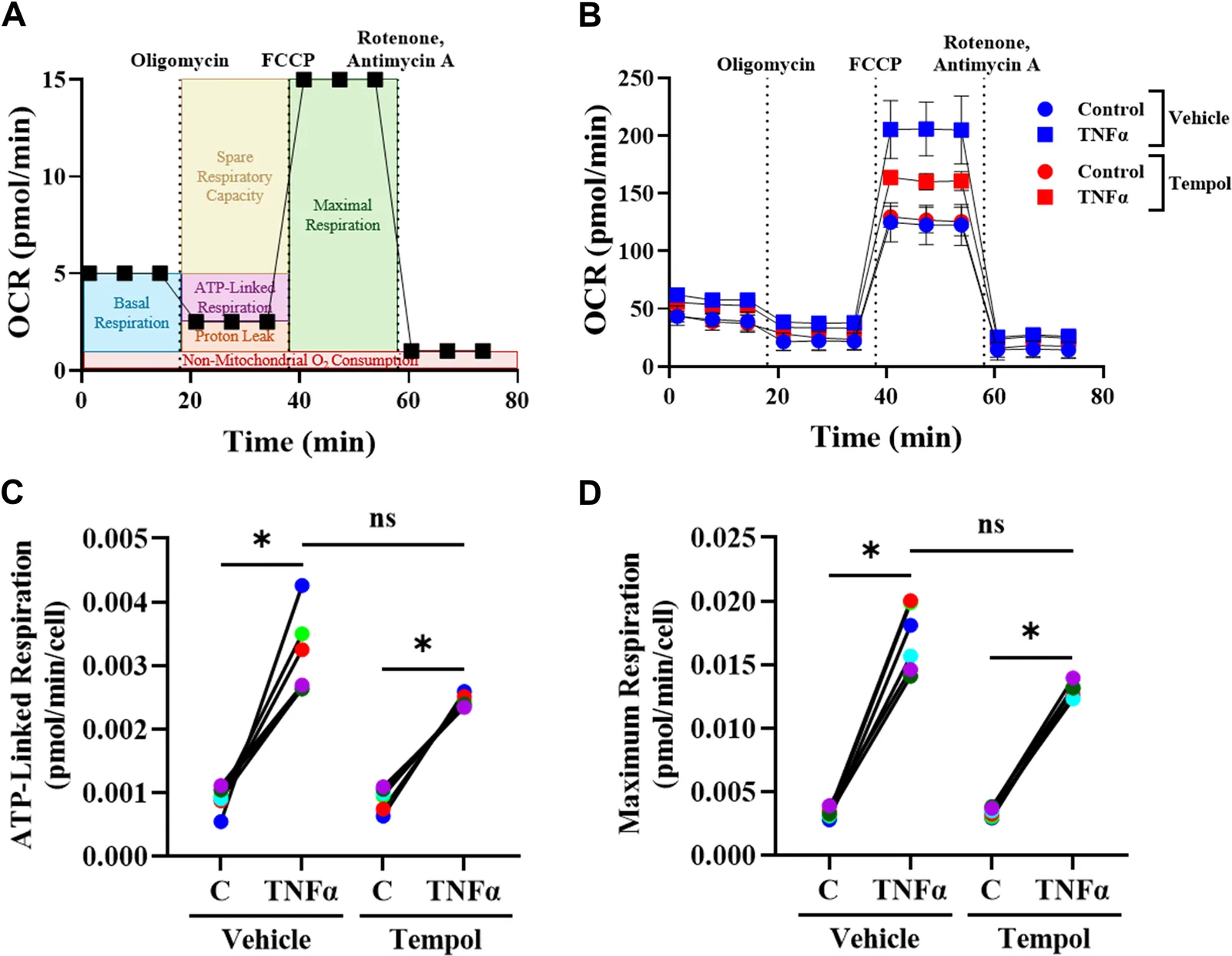
Tempol decreases the TNFα-induced increase in OCR per cell. *A*: seahorse mitochondrial stress test was performed to assess maximum respiration and ATP-linked respiration. *B*: OCR measurements were obtained from treated and control hASM cells and normalized to the total cell count. Blue symbols represent vehicle-treated hASM cells, and red symbols represent tempol-treated hASM cells. Data are presented as means ± SD in a nonlinear regression model (curve fit analysis). *C*: TNFα increased ATP-linked respiration when compared with controls (*P < 0.05). Pretreatment with tempol decreased the TNFα-induced increase in ATP-linked respiration, but not significantly (*P* = 0.052). *D*: TNFα increased maximal OCR when compared with controls (*P < 0.05). Pretreatment with tempol decreased TNFα-mediated increase in maximal OCR, but not significantly (*P* = 0.065). Each color represents results from one patient sample. Data are presented as means ± SD in scatter plot. Statistical analyses were based on measurements from *n* = 6 (3 females and 3 males; 4 wells/patient) patients using mixed-effects analysis. hASM, human airway smooth muscle; OCR, O_2_ consumption rate.

**Figure 8.**
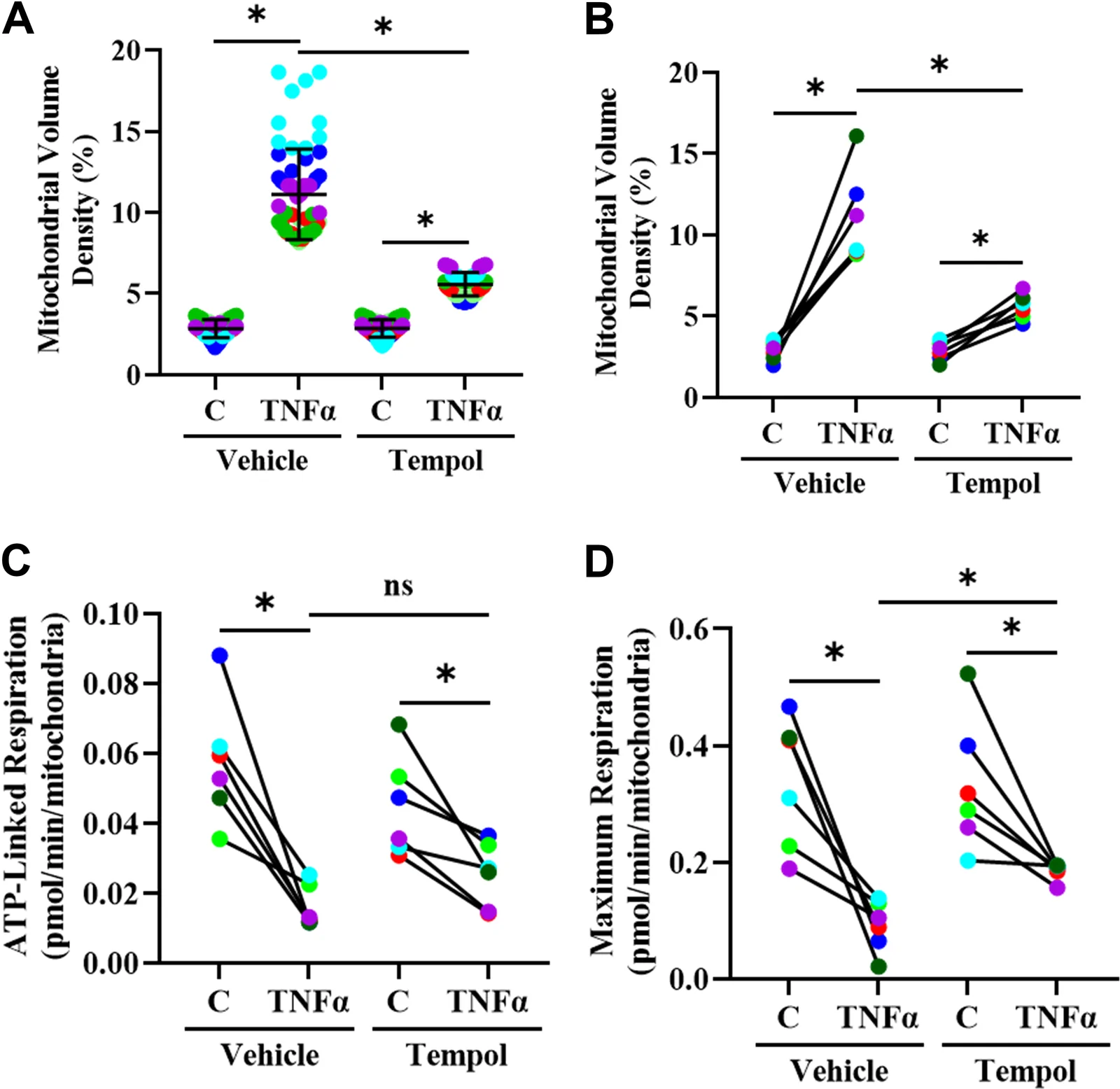
Tempol increases the TNFα-induced increase in OCR per mitochondrion. Mitochondrial volume density was measured using three-dimensional (3-D) reconstructed Z-stack images of fluorescently labeled mitochondria using ImageJ in hASM cells treated with TNFα (20 ng/mL, 6 h), TNFα (20 ng/mL, 6 h) with tempol (500 μM, 30 min pretreatment), and untreated (control). *A* and* B*: mitochondrial volume density was increased in TNFα-treated cells when compared with controls (*P < 0.05), and pretreatment with tempol significantly decreases mitochondrial volume density (*P < 0.05). Scatter plot (*A*) and symbols and line plot (*B*) show mitochondrial volume density from 6 patients (3 females and 3 males; 10 cells/patient) per treatment group. OCR values per patient were normalized to their respective mean mitochondrial volume. *C*: ATP-linked respiration per mitochondrion significantly decreased in TNFα-treated hASM cells when compared with vehicle-treated controls (*P < 0.05). Pretreatment with tempol showed no significant change in the TNFα-induced decrease in ATP-linked respiration per mitochondrion. *D*: maximal OCR per mitochondrion significantly decreased in TNFα-treated hASM cells when compared with vehicle-treated controls (*P < 0.05). Pretreatment with tempol significantly increased TNFα-mediated decrease in maximal OCR per mitochondrion. Each color represents results from one patient sample. Data are presented as means ± SD in scatter plot. Statistical analyses were based on measurements from *n* = 6 (3 females and 3 males) patients using mixed-effects analysis. hASM, human airway smooth muscle; OCR, O_2_ consumption rate.

A mitochondrial stress test was performed to assess overall mitochondrial respiration, maximal OCR, and ATP-linked respiration, and normalized to cell count (Fig. 7*A*). Compared to vehicle-treated hASM cells, TNFα significantly increased mitochondrial OCR per cell, which was mitigated by tempol (Fig. 7*B*). Although not statistically significant, Tempol decreased the TNFα-induced increase in ATP-linked respiration per cell by ∼1.2-fold (Fig. 7*C*) and maximal OCR per cell by ∼1.6-fold (Fig. 7*D*). No significant difference was observed between vehicle-treated hASM cells and cells treated only with tempol. Mitochondrial volume density was measured using MitoTracker-labeled mitochondria within hASM cells. Consistent with previous studies (18, 30, 31), mitochondrial volume density was significantly increased by ∼4-fold in hASM cells treated with TNFα compared to vehicle-treated control hASM cells (Fig. 8, *A* and *B*; *P* < 0.05). Pretreatment with tempol significantly reduced the TNFα-induced increase in mitochondrial volume density compared to cells treated only with TNFα (Fig. 8, *A* and *B*; *P* < 0.05). When OCR per patient was normalized to their respective mean mitochondrial volume, with TNFα exposure, both ATP-linked respiration per mitochondrion and maximal OCR per mitochondrion significantly decreased, consistent with previous studies (18, 30, 31). Although tempol had no significant effect on the TNFα-induced decrease in ATP-linked respiration per mitochondrion (Fig. 8*C*), pretreatment with tempol significantly increased maximal OCR per mitochondrion by ∼2-fold (Fig. 8*D*; *P* < 0.05). No significant difference was observed between vehicle-treated hASM cells and cells treated only with tempol.

## DISCUSSION

The results of the present study support the hypothesis that cellular ROS formation is a critical mediator of TNFα-induced activation of the pIRE1α^S724^/XBP1s ER stress pathway and subsequent fragmentation of mitochondria in hASM cells. The key findings of the study are *1*) TNFα significantly increases cellular as well as mitochondrial ROS formation; *2*) TNFα activates the pIRE1α^S724^/XBP1s-mediated ER stress pathway; *3*) TNFα- induced activation of pIRE1α^S724^/XBP1s ER stress pathway results in downstream expression of CDK1 and CDK5, which mediate phosphorylation of pDRP1^S616^ and mitochondrial fragmentation; *4*) scavenging ROS with tempol attenuates TNFα-induced activation of pIRE1α^S724^/XBP1s ER stress pathway; *5*) tempol suppresses TNFα-induced expression of CDK1 and CDK5, phosphorylation of pDRP1^S616^, and mitochondrial fragmentation; *6*) tempol increased maximal respiration when normalized to mitochondrial volume, which signifies that tempol improves the intrinsic respiratory capacity and functional efficiency of individual mitochondria without altering overall cellular oxygen consumption. Together, these findings identify a mechanistic link between TNFα-induced ROS formation, activation of the pIRE1α^S724^/XBP1s ER stress pathway, and mitochondrial fragmentation in hASM cells.

### TNFα Induces ROS Formation in hASM Cells

The results of the present study are consistent with the previous findings from our laboratory, showing that TNFα exposure increases ROS formation in hASM cells (30). Previous studies from our laboratory showed that in porcine ASM, TNFα increases force generation and ATP consumption, triggering increased O_2_ consumption (6, 7). In hASM cells, we found that in response to TNFα, mitochondrial volume density increases (10, 26, 30) and maximal O_2_ consumption rate increases (31), which can result in higher ROS production. During O_2_ consumption, mitochondrial complexes I and III in the electron transport chain are major sites of ROS production. Studies indicate that TNFα can trigger an increase in mitochondrial ROS production via several mechanisms, including retrograde electron transport in complex I (30, 41), increased mitochondrial membrane permeability, or dysregulated electron transport chain activity (42–47). Although increased O_2_ consumption and mitochondrial damage serve as a sustained source of ROS production, other studies have reported that activation of NAPDH oxidase (NOX2 and NOX4) may also serve as an early source of TNFα-induced ROS in hASM cells (48–50). A study by Hollins et al. (50) demonstrated that TNFα treatment upregulates NOX4 expression in hASM cells from subjects with chronic obstructive pulmonary disease (COPD), and NOX4 inhibition resulted in complete abrogation of oxidant-induced ROS generation.

In this study, we demonstrated that TNFα treatment significantly increased both DCFDA and MitoSox fluorescence, indicating an overall elevation in cellular oxidative stress accompanied by enhanced mitochondrial superoxide production. The 70% greater increase observed in MitoSox fluorescence relative to DCFDA suggests that mitochondria represent a major source of ROS following TNFα treatment (Fig. 3, *F* and *G*). In contrast, the DCFDA signal reflects the overall intracellular oxidative environment and may include ROS derived from nonmitochondrial sources such as NADPH oxidases and peroxisomes. Tempol mitigated ROS formation from both sources; however, the effect of tempol was more prominent, reducing mitochondrial ROS. The ROS signals coming from control and tempol-treated cells were primarily nonmitochondrial (∼20%–30% is mitochondrial).

### ROS Underlies Protein Unfolding and Induces ER Stress

Consistent with ROS-induced protein unfolding, we previously found that treatment with the chemical chaperone 4-PBA mitigates TNFα-induced ER stress in hASM cells (16). ROS can mediate protein structural changes and unfolding via multiple mechanisms. For example, ROS can alter amino acid structures via oxidation, causing covalent modifications to amino acid side chains (51–54). This oxidative damage mainly targets the sulfur-containing and aromatic amino acids, such as cysteine, methionine, and tryptophan, alters amino acid chemistry, disrupts protein folding, and causes structural denaturation, resulting in the loss of biological activity (51, 55, 56). By changing the redox balance of the cell, ROS shifts the cellular microenvironment away from that favoring proper protein folding (57, 58). Elevated ROS in the ER can also alter calcium homeostasis within a cell (59–61), which can impair the function of molecular chaperones (62–64), leading to further accumulation of unfolded proteins within the ER lumen (65). As unfolded proteins accumulate in the ER, this triggers an ER stress response (66–69).

With respect to the TNFα-induced ER stress response in hASM cells, we previously showed that tunicamycin, a mixture of nucleoside antibiotics, can activate all three ER stress pathways mediated by IRE1α, PERK, and ATF6 (9). However, in hASM cells, TNFα triggers only the IRE1α-mediated ER stress pathway, without activating PERK- or ATF6-mediated pathways (9, 16–18). The mechanism underlying the selective activation of the IRE1α ER stress pathway by TNFα is not known. However, it is possible that BiP-binding affinity to IRE1α is lower than that for PERK or ATF6. Regardless, BiP dissociation from IRE1α triggers oligomerization of IRE1α, activating the kinase domain leading to autophosphorylation of pIRE1α^S724^ (70, 71). The results of the present study are consistent with our previous observations showing an increase in total IRE1α level after 6 h of TNFα treatment (18). The TNFα-induced increase in IRE1α was mitigated by tempol, consistent with TNFα-induced ROS formation being involved in the activation of the IRE1α/XBP1s ER stress pathway.

### ROS Induces XBP1-Mediated Transcriptional Activation of Target Genes

We previously demonstrated that in hASM cells, TNFα-induced autophosphorylation of pIRE1α^S724^ triggers the activation of its cytoplasmic endoribonuclease (RNase) activity, mediating alternative splicing of *XBP1* mRNA (9, 16–18). In the present study, we extended this finding by showing that tempol-mediated scavenging of ROS prevented TNFα-induced splicing of XBP1s*. XBP1u* codes for an ∼29 kDa inactive protein, which is eventually targeted for proteasomal degradation. *XBP1s* codes for an ∼50 kDa active transcription factor that translocates to the nucleus and binds to the unfolded protein response element (UPRE sequence TGACGTGG/A) present in the promoter region of its target genes, activating transcription (72–76). Consistent with results of a previous study, we found that XBP1s targets CDK1 and CDK5 (18). In addition to regulating the cell cycle (17), CDK1 and CDK5 also play a significant role in mitochondrial fragmentation by phosphorylating pDRP1^S616^. In support, we found that inhibition of XBP1u to XBP1s splicing by pharmacological and genetic inhibitors reduced CDK1 and CDK5 expression (17).

### ROS Induces Mitochondrial Fragmentation

Results of the present study indicate that the TNFα-induced increase in CDK1 and CDK5 expression led to an increase in pDRP1^S616^ phosphorylation and mitochondrial fragmentation, consistent with the results of a previous study (18). In the present study, we extended these results by demonstrating that ROS scavenging inhibited CDK1 and CDK5 expression, pDRP1^S616^ phosphorylation, and mitochondrial fragmentation in hASM cells. ROS plays an important role in regulating mitochondrial dynamics (77–79). Elevated ROS levels can disrupt the balance between mitochondrial fusion and fission, leading to increased mitochondrial fragmentation (77–79). Excess ROS promotes the phosphorylation of pDRP1^S616^ and activation of DRP1 recruitment to mitochondria, which drives mitochondrial fragmentation (80–86). As a result, mitochondria transition from elongated interconnected networks to shorter, fragmented structures. This ROS-induced mitochondrial fragmentation is commonly observed under conditions of cellular stress and is often associated with mitochondrial dysfunction, reduced ATP production, and the initiation of mitophagy or apoptotic pathways (87–91). Therefore, elevated ROS levels not only act as damaging agents but also function as signaling molecules that modulate mitochondrial morphology and cellular fate.

### ROS-Mediated Changes in Mitochondrial Function

In the present study, OCR was measured in hASM cells and normalized to cell number and mitochondrial volume density. Consistent with our previous finding (31), TNFα treatment increased ATP-linked respiration and maximal respiration per cell after 6 h, indicating an overall increase in cellular respiratory activity. However, in the present study and in a previous study (31), we found that both OCR and mitochondrial volume density increased following TNFα treatment. When TNFα-induced changes in OCR were normalized to the increase in mitochondrial volume density, OCR per mitochondrial volume was significantly reduced. In the present study, we extended this finding by demonstrating that tempol-mediated scavenging of ROS does not significantly prevent the TNFα-induced increase in ATP-linked respiration and maximal respiration when normalized to cell number. When normalized for mitochondrial volume, tempol had no effect on ATP-linked respiration. However, tempol increased maximal respiration when normalized to mitochondrial volume, which signifies that tempol improves the intrinsic respiratory capacity and functional efficiency of individual mitochondria without altering overall cellular oxygen consumption.

### Conclusions

Findings of the present study together with those of previous studies support our hypothesis that in hASM cells TNFα induces production of cellular ROS, which can trigger the accumulation of unfolded proteins in the ER. This accumulation of unfolded proteins selectively activates the pIRE1α^S724^/XBP1s ER stress pathway, promoting downstream transcriptional activation of CDK1 and CDK5 protein expression that mediates pDRP1^S616^ phosphorylation. An increase in pDRP1^S616^ underlies increased fragmentation of mitochondria. ROS scavenging via tempol decreases cellular ROS, reduces TNFα-mediated activation of pIRE1α^S724^/XBP1s ER stress pathway and downstream CDK1/5-mediated phosphorylation of pDRP1^S616^, and prevents mitochondrial fragmentation and improves the intrinsic respiratory capacity and functional efficiency of individual mitochondria.

### Clinical Significance

The role of ROS formation and potential impact of oxidative stress in airway diseases such as asthma or chronic obstructive pulmonary disease is well recognized (92–94). In the present study, we showed that the ROS scavenger tempol is effective in mitigating TNFα-induced pIRE1α^S724^ and XBP1s and subsequent fragmentation of mitochondria in hASM cells, showing potential future therapeutic application. However, antioxidants and ROS scavenging strategies have proved to be challenging, with potential serious side effects on patients, and further studies will be needed. Chronic and excessive fragmentation of mitochondria often leads to impaired mitochondrial function and ATP production and release of mtDNA into the cytosol, triggering localized inflammation and cell death (78, 95–98). Potential therapeutics targeting mitochondrial fragmentation and restoring the balance between mitochondrial fragmentation and fusion will be beneficial to treat diseases characterized by dysfunction of mitochondria.

## Supplementary Material

Supplemental Material

## Data Availability

All data are presented in the main manuscript.
